# Platelet Phenotype Analysis of COVID-19 Patients Reveals Progressive Changes in the Activation of Integrin αIIbβ3, F13A1, the SARS-CoV-2 Target EIF4A1 and Annexin A5

**DOI:** 10.3389/fcvm.2021.779073

**Published:** 2021-11-11

**Authors:** Huriye Ercan, Waltraud Cornelia Schrottmaier, Anita Pirabe, Anna Schmuckenschlager, David Pereyra, Jonas Santol, Erich Pawelka, Marianna T. Traugott, Christian Schörgenhofer, Tamara Seitz, Mario Karolyi, Jae-Won Yang, Bernd Jilma, Alexander Zoufaly, Alice Assinger, Maria Zellner

**Affiliations:** ^1^Center for Physiology and Pharmacology, Institute of Vascular Biology and Thrombosis Research, Medical University of Vienna, Vienna, Austria; ^2^Division of Visceral Surgery, Department of General Surgery, General Hospital Vienna, Medical University of Vienna, Vienna, Austria; ^3^Department of Medicine IV, Clinic Favoriten, Vienna, Austria; ^4^Department of Clinical Pharmacology, Medical University of Vienna, General Hospital Vienna, Vienna, Austria; ^5^Center for Physiology and Pharmacology, Institute of Pharmacology, Medical University of Vienna, Vienna, Austria

**Keywords:** COVID-19, thrombosis, platelets, integrin αIIbβ3, coagulation factor XIII (FXIII, F13A1), antiphospholipid syndrome, annexin A5, eukaryotic initiation factor (EIF4A1)

## Abstract

**Background:** The fatal consequences of an infection with severe acute respiratory syndrome coronavirus 2 are not only caused by severe pneumonia, but also by thrombosis. Platelets are important regulators of thrombosis, but their involvement in the pathogenesis of COVID-19 is largely unknown. The aim of this study was to determine their functional and biochemical profile in patients with COVID-19 in dependence of mortality within 5-days after hospitalization.

**Methods:** The COVID-19-related platelet phenotype was examined by analyzing their basal activation state via integrin αIIbβ3 activation using flow cytometry and the proteome by unbiased two-dimensional differential in-gel fluorescence electrophoresis. In total we monitored 98 surviving and 12 non-surviving COVID-19 patients over 5 days of hospital stay and compared them to healthy controls (*n* = 12).

**Results:** Over the observation period the level of basal αIIbβ3 activation on platelets from non-surviving COVID-19 patients decreased compared to survivors. In line with this finding, proteomic analysis revealed a decrease in the total amount of integrin αIIb (ITGA2B), a subunit of αIIbβ3, in COVID-19 patients compared to healthy controls; the decline was even more pronounced for the non-survivors. Consumption of the fibrin-stabilizing factor coagulation factor XIIIA (F13A1) was higher in platelets from COVID-19 patients and tended to be higher in non-survivors; plasma concentrations of the latter also differed significantly. Depending on COVID-19 disease status and mortality, increased amounts of annexin A5 (ANXA5), eukaryotic initiation factor 4A-I (EIF4A1), and transaldolase (TALDO1) were found in the platelet proteome and also correlated with the nasopharyngeal viral load. Dysregulation of these proteins may play a role for virus replication. ANXA5 has also been identified as an autoantigen of the antiphospholipid syndrome, which is common in COVID-19 patients. Finally, the levels of two different protein disulfide isomerases, P4HB and PDIA6, which support thrombosis, were increased in the platelets of COVID-19 patients.

**Conclusion:** Platelets from COVID-19 patients showed significant changes in the activation phenotype, in the processing of the final coagulation factor F13A1 and the phospholipid-binding protein ANXA5 compared to healthy subjects. Additionally, these results demonstrate specific alterations in platelets during COVID-19, which are significantly linked to fatal outcome.

## Graphical Abstract

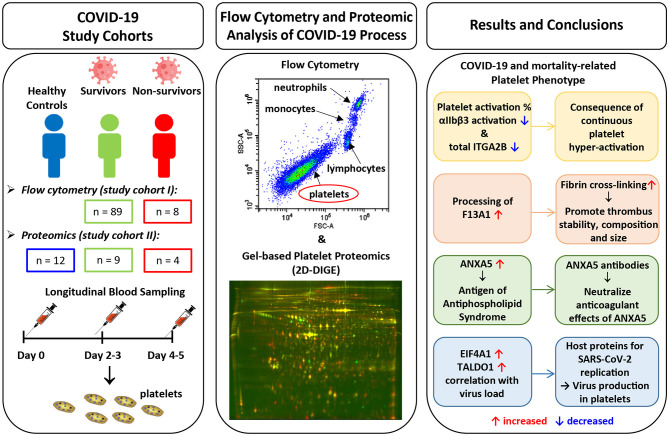

## Introduction

Coronavirus disease 2019 (COVID-19), caused by severe acute respiratory syndrome corona virus 2(SARS-CoV-2) infection, is characterized by variable clinical features and degrees of severity, ranging from asymptomatic, mild influenza-like symptoms to life-threatening respiratory distress, and multiple organ failure (1–3). Innate immune responses against SARS-CoV-2 lead to activation of the coagulation cascade (4), with presence of microthrombi not only in pulmonary tissue of deceased COVID-19 patients, but also in distant organs like heart and kidney (5–7). Hence, COVID-19 shows features of an immuno-thrombotic disease (8–10), with clinical thrombosis incidences reaching up to 40% among COVID-19 patients (11–14), particularly in critically ill patients requiring intensive care (15–17). Even with controlled thromboprophylaxis, VTE occurred in 27% of COVID-19 patients in the medical ward unit and 76% in the intensive care units (18). Notably, thromboembolisms were observed both at arterial and venous sites indicating a derangement of both platelet mediated and plasmatic coagulation (12, 19–22).

The role of platelets in COVID-19 is still incompletely understood. Thrombocytopenia was found to be associated with disease severity (23–26) and platelet apoptosis observed in COVID-19 patients requiring intensive care unit treatment (27). In line with these findings, severely ill COVID-19 patients display elevated markers of platelet activation (9, 26, 28–30) and elevated plasma levels of platelet activation markers (31, 32).

It is also noteworthy that platelet exhaustion and hypo-responsiveness of platelets have been observed in COVID-19 patients. Platelets of COVID-19 patients show hypo-reactivity in response to *in-vitro* stimulation (9, 29, 30, 32, 33), indicating prior platelet hyper-activation and resulting hypo-responsiveness in COVID-19 patients (34). However, little is known about more detailed molecular changes in platelets in the context of SARS-CoV-2 infection and the course of the disease. Therefore, the aim of this study was to decipher specific changes in platelet function associated with COVID-19 as well as between COVID-19 survivors and non-survivors. We analyzed platelet activation status by means of flow cytometry and platelet proteome using 2D-DIGE technology over a period of 5 days in a cohort of hospitalized COVID-19 patients.

## Materials and Methods

### Study Design

In total 110 patients with COVID-19 (98 survivors and 12 non-survivors) admitted to the central COVID-19 hospital Clinic Favoriten, Vienna, Austria, between April and November 2020 were included in this study. Flow cytometry analysis was performed on the first 97 patients enrolled and platelet proteome analysis was performed on the following 13 patients. Blood was taken upon study entry (day 0), on day two to three (day 2–3) and on day four to five (day 4–5) after enrollment. Outcome data was available for all patients at the time of analysis. All patients gave written informed consent and the study was conducted in accordance with the Declaration of Helsinki. The collection of data was part of the ACOVACT study (ClinicalTrials.gov: NCT04351724) approved by the local ethics committee (EK1315/2020). This study was approved by the Ethics Committee of the Medical University of Vienna in accordance with the Declaration of Helsinki (EK1548/2020). Patient demographics including comorbidities and use of medication were recorded. Routine laboratory analysis was performed upon admission and every second day afterwards. Nasopharyngeal swabs and quantitative polymerase chain reaction (qPCR) for SARS-CoV-2 were performed according to the Charité protocol (35). Peripheral vein blood was also collected from 12 SARS-CoV-2 negative healthy volunteers recruited among the research staff of the institute (median age, 61 years; age range, 44–63; 58% male; Supplementary Table 1).

### Blood Collection, Washed Platelet, and Plasma Isolation

For platelet isolation, blood was drawn from an antecubital vein into 3.5 mL vacuum tubes containing 0.129 mM trisodium citrate as anticoagulant (Greiner Bio-One, Kremsmünster, Austria). To obtain platelet rich plasma (PRP), two 1 mL aliquots of citrated blood in 1.5 mL tubes were centrifuged [8 min, 67 g, room temperature (RT)] and the supernatant PRP was pooled into a fresh 1.5 mL tube. Platelets were pelleted (2 min, 2,000 g, RT) in the presence of 0.8 μM PGI_2_ (Sigma-Aldrich, St. Louis, MO, USA) and washed once in phosphate-buffered saline (w/o: Ca^2+^ and Mg^2+^) containing PGI_2_ (0.8 μM). The supernatant was carefully discarded and the platelet pellet was frozen at −80°C until further processing.

For plasma preparation citrated blood was centrifuged (10 min, 1,000 g, 4°C) to separate the cellular fraction and the plasma supernatant, which was subsequently cleared of debris by a second centrifugation step (10 min, 10,000 g) and stored at −80°C.

### Flow Cytometric Platelet Analysis

Citrated whole blood obtained at day 0, day 2–3, and day 4–5 was stained with PerCP-labeled anti-CD42b (1:75, Biolegend) and FITC-labeled PAC-1 antibodies (1:40, BD Biosciences) for 20 min at RT in the dark. Platelets were fixed and erythrocytes lysed by addition of 1-step Fix/Lyse solution (eBioscience). Samples were diluted with PBS and analyzed using a Cytoflex S cytometer and CytExpert 2.4 software (both Beckman Coulter). Platelets were specifically gated by the specific signal from the antibody against CD42b (glycoprotein Ib—receptor for von Willebrand factor) with a PerCP-labeled anti-CD42b (1:75, Biolegend). Accordingly only flow cytometry singlet events in the size and granularity (FSC and SSC) of platelets and positive for the CD42b signal were gated for PAC-1 binding. PAC-1 binding (FITC-labeled PAC-1 antibody; 1:40, BD Biosciences) was then quantified as % positive of CD42 + events. Gate location for PAC-1 was confirmed with activated platelets (Supplementary Figure 1).

### Platelet Preparation for Two-Dimensional Fluorescence Differential Gel Electrophoresis (2D-DIGE) Analysis

The frozen platelet protein pellets were resolubilized in urea-sample buffer (7 M urea, 2 M thiourea, 4% CHAPS, 20 mM Tris-HCl pH 8.68) and incubated for 1 h at RT under agitation (800 rpm). Protein quantitation of individual samples was done in duplicate with a Coomassie brilliant blue protein assay kit (Pierce, Thermo Scientific, Rockford, IL, USA). The internal standard (IS) was prepared by pooling equal protein amounts of all included samples. Platelet protein samples and IS were aliquoted and stored at −80°C.

### Platelet Proteome Analysis by 2D-DIGE

Proteins were labeled with fluorescent cyanine dyes (5 pmol of CyDyes per μg of protein; Cytivia, Hoegaarden, Belgium) according to our previous publication (36). The IS was always labeled using Cy2, while Cy3 and Cy5 were used alternately for study samples. Briefly, IPG-Dry-Strips (24 cm, pH 4–7, Cytivia, Hoegaarden, Belgium) were rehydrated for 11 h with 450 μL rehydration buffer (7 M urea, 2 M thiourea, 70 mM DTT, 0.5% pH 4–7 ampholyte; Serva, Heidelberg, Germany) mixed with a total of 30 μg (2 × 10 μg sample + 1 × 10 μg IS) of alternatively Cy-labeled sample. Isoelectric focusing (IEF) was performed on a Protean I12 IEF unit (Biorad, Hercules, California,) until 30 kVh was reached.

After IEF, the strips were first equilibrated with gentle shaking in 12.5 mL of equilibration buffer 1 (1% DTT, 50 mM Tris-HCl pH 8.68, 6 M Urea, 30% glycerol and 2% SDS) for 20 min followed by incubation in equilibration buffer 2 (2.5% iodoacetamide, 50 mM Tris-HCl pH 8.68, 6 M Urea, 30% glycerol and 2% SDS) for 15 min. The IPG-strips were transferred on 11.5% acrylamide gel (26 × 20 cm, 1 mm gel) and sealed with low melting agarose sealing solution (375 mM Tris-HCl pH 8.68, 1% SDS, 0.5% agarose). SDS-PAGE was performed using an Ettan DALTsix electrophoresis chamber (GE Healthcare, Uppsala, Sweden) under the following conditions: 35 V for 1 h, 50 V for 1.5 h and finally 110 V for 16.5 h at 10°C.

### 2D-DIGE Image Analysis

For protein spot detection, 2D-DIGE gels were scanned at 489, 550, and 649 nm corresponding to the three different excitation wavelengths of the CyDyes and imaged with a resolution of 100 μm using a Typhoon 9410 Scanner (GE Healthcare, Uppsala, Sweden). Gel images were analyzed *via* the DeCyder™ software (version 7.2, GE Healthcare, Uppsala, Sweden). Spots were matched to a master 2D-DIGE gel (a representative pH 4-7 platelet protein map of the IS images). On average, 400 protein spots were matched manually to the master gel using the DeCyder™ software. Afterwards an automatic spot match was used which achieved an average of 2100 matched spots per gel. Detailed information about the image analysis was published by Winkler et al. (37). The standardized abundance (SA) of every protein spot was calculated by the DeCyder™ software with two normalization steps. Since we only carried out one washing step for platelet isolation due to the COVID-19-related safety measures, we included an additional normalization step using a geometric mean of eight low biological variable platelet proteins (YWHAE, YWHAZ, YWHAH, TPM4, ATP5F1B, GNB1, GRB2, PRDX6; Supplementary Table 2; Supplementary Figures 2, 3), which we previously identified in the proteomic database of washed platelets (36) and gel-filtrated platelets (38). This normalization step ensured that the respective plasma contamination does not affect the exact quantification of the platelet proteins.

### Protein Identification *via* Mass Spectrometry

For MS-based identifications, 250 μg unlabelled proteins were separated by the same 2D-DIGE equipment that was used for the fluorescently-labeled samples described samples above. Proteins were visualized by MS-compatible silver staining (39). Protein spots of interest were excised manually from the gels, de-stained, disulfide was reduced and afterwards derivatized with iodoacetamide and the proteins were tryptically digested. An electrospray ionization (ESI)-quadrupole-time-of-flight (QTOF; Compact, Bruker) coupled with an Ultimate 3000 nano-HPLC system (Dionex) was used for LC-MS/MS data acquisition. A PepMap100 C-18 trap column (300 μm × 5 mm) and PepMap100 C-18 analytic column (75 μm × 250 mm) were used for reverse phase (RP) chromatographic separation with a flow rate of 500 nl/min. The two buffers used for the RP chromatography were 0.1% formic acid/water and 0.08% formic acid/80% acetonitrile/water with gradient condition for 90 min. Eluted peptides were then directly sprayed into the mass spectrometer and the MS/MS spectra were interpreted with the Mascot search engine (version 2.7.0, Matrix Science, London, UK) against Swissprot database (564,277 sequences, released in January 2021) and the taxonomy was restricted to homo sapiens (human; 20,397 sequences). The search parameters were used with a mass tolerance of 10 ppm and an MS/MS tolerance of 0.1 Da. Carbamidomethylation (Cys), oxidation (Met), phosphorylation (Ser, Thr, and Tyr), acetylation (Lys and N-term), and deamidation (Asn and Gln) were allowed with 2 missing cleavage sites. The Mascot cut-off score was set to 15 and proteins identified with two or more peptides were considered (40).

### One and Two-Dimensional Western Blot Analysis

For one-dimensional Western blot (1-D WB), a total of 12 μg platelet protein were mixed with a sample buffer (150 mM Tris-HCl pH 8.68, 7.5% SDS, 37.5% glycerol, bromine phenol blue, 125 mM DTT) to obtain a final volume of 20 μL. Samples were boiled for 4 min at 95°C and centrifuged for 3 min at 20,000 g. Thereafter, the samples were separated in a 11.5% SDS gel (50 V, 20 min and 100 V, 150 min) and blotted (75 V, 120 min) on a polyvinylidene difluoride membrane (PVDF; FluoroTrans® W, Pall, East Hills, NY, USA).

For two-dimensional Western blot (2-D WB) analysis, 30 μg Cy2-labeld platelet proteins were separated by IEF on a 24 cm pH 4–7 IPG strip as described for 2D-DIGE gels, and subsequently transferred onto a PVDF membrane (75 V, 90 min). The membranes were blocked with 5% non-fat dry milk (BioRad, Hercules, CA, USA) in 1x PBS containing 0.3% Tween-20 (PBS-T) over night at 4°C under gentle shaking. Membranes were washed (PBS-T, 3x 5 min) and incubated with monoclonal anti-Factor F13A1 antibody (1:250 in PBS-T containing 3% non-fat dry milk; ab1834; Abcam, USA) for 2 h at RT (180 rpm). After washing (PBS-T, 3x 5 min), the membranes were incubated with a horseradish peroxidase (HRP)- conjugated secondary antibody (1:20,000 in PBS-T containing 3% non-fat dry milk) for 1.5 h in the dark at RT (65 rpm). Membranes were washed again (2x 5 min in PBS-T, 1x 5 min in PBS) and the HRP signal was detected using an Enhanced Chemiluminescent substrate (FluorChem® HD2, Alpha Innotech, CA, USA).

### Measurement of Haemostatic Biomarkers in Plasma

F13A1 and D-dimer were assessed using LEGENDplex Human Fibrinolysis Panel Kit (BioLeged) according to manufacturer's instruction, measured on a Cytoflex S cytometer and analyzed by LEGENDplex v8.0 software (BioLegend). This multiplex bead-based assay works with beads of differential size and internal fluorescence intensities. Each bead set is conjugated with a specific antibody on its surface and serves as the capture beads for that particular analyte. As with the ELISA system, the specific analyte is then made detectable for flow cytometry with the respective specific detection antibody.

### Biological Pathway Analysis

To get an initial insight into the biological function of the newly revealed COVID-19-related platelet proteins, a protein-protein interaction network analysis was performed. The data source was the protein query of the STRING database (Version 11.0b) (41), with the following settings (active interaction sources: experiments and databases; score = 0.4; maximal additional interactors = 0). For the functional enrichment, the Gene Ontology Biological Processes and KEEG pathway analyses were used for the PPI networks with a specific color for each biological process and KEGG pathway. The STRING Version 11.0b was used.

### Statistics

For explorative statistical analysis, only 2D-DIGE protein image spots were included which could be matched by the IS spot map with more than 95% of all 2-D platelet proteome maps of this study. This quality selection limits the resulting protein spots to 420 out of an average of 2100 spot events matched with the master gel. One-way analysis of variance (ANOVA) was calculated for these 420 reliably matched spots between the five study groups (COVID-19 survivors day 0, COVID-19 non-survivors day 0, COVID-19 survivors day 4–5, COVID-19 non-survivors day 4–5, and healthy controls). Significant differences between control group and COVID-19 patients and between patients with different outcome were analyzed by planned contrasts analysis in SPSS Statistics 25 (SPSS Inc, Chicago, USA). Graphs were created with GraphPad Prism 7 (GraphPad Software, Inc. San Diego California, USA).

## Results

### Patient Characteristics of the Two Study Cohorts

To determine platelet-specific differences between COVID-19 survivors, non-survivors and healthy controls we included 89 surviving and 8 non-surviving COVID-19 patients for flow cytometric analysis of basal platelet activation (study cohort I) (Tables 1A, 2A). For the platelet proteomics analysis, 9 additional surviving and 4 non-surviving COVID-19 patients as well as 12 healthy controls were included (study cohort II) (Tables 1B, 2B). Detailed characteristics of the COVID-19 patient demographics are presented in Tables 1, 2. The median age of the healthy controls was 61 years (Demographics: Supplementary Table 1).

**Table 1:** Patient demographics flow cytometric study cohort I **(A)** and proteomics study cohort II **(B)**.

**A: Study cohort I**					
	**Missing data**	**All** **(*****n =*** **97)**	**Survivors** **(*****n =*** **89)**	**Non-survivors** **(*****n =*** **8)**	
**Parameter**	***n***	***n*** **(%)** **Median (IQR)**	***n*** **(%)** **Median (IQR)**	***n*** **(%)** **Median (IQR)**	***P*** -value^*^
**Sex**	–				0.355
Male		63 (65)	59 (66)	4 (50)	
Female		34 (35)	30 (34)	4 (50)	
**Age (years)**	–	61 (49–77)	59 (69–73)	83 (79–86)	**< 0.001**
**Comorbidities**					
Current smoker	37	5 (8.3)	5 (8.6)	0 (0.0)	0.665
Obesity (BMI > 25)	21	56 (73.7)	53 (73.6)	3 (75.0)	0.951
Diabetes type II	–	25 (25.8)	20 (22.5)	5 (62.5)	**0.013**
Hypertension	1	54 (56.3)	46 (52.3)	8 (100.0)	**0.009**
Cardiovascular disease (any)	–	26 (26.8)	20 (22.5)	6 (75.0)	**0.001**
Coronary heart disease	–	13 (13.4)	9 (10.1)	4 (50.0)	**0.002**
Chronic heart failure	–	9 (9.3)	6 (6.7)	3 (37.5)	**0.004**
Atrial fibrillation	–	11 (11.3)	8 (9.0)	3 (37.5)	**0.015**
Peripheral arterial disease	–	4 (4.1)	2 (2.2)	2 (25.0)	**0.002**
Chronic obstructive pulmonary disease	–	10 (10.3)	10 (11.2)	0 (0.0)	0.317
Asthma	–	5 (5.2)	4 (4.5)	1 (12.5)	0.337
Hypo-/Hyperthyroidism	1	9 (9.4)	8 (9.1)	1 (12.5)	0.752
Chronic renal insufficiency	–	13 (13.4)	11 (12.4)	2 (25.0)	0.315
Chronic liver disease	1	4 (4.2)	3 (3.4)	1 (14.3)	0.164
Malignancy	–	8 (8.2)	8 (9.0)	0 (0.0)	0.376
**Medication (anti-platelet/anticoagulation)**					
Anti-platelet therapy	–	15 (15.5)	11 (12.4)	4 (50.0)	**0.005**
Anticoagulation therapy	–	94 (96.9)	86 (96.6)	8 (100.0)	0.598
**COVID-19 classification at admission^†^**	–				0.304
Asymptomatic/mild		15 (15.5)	14 (15.7)	1 (12.5)	
Moderate		46 (47.4)	44 (49.4)	2 (25.0)	
Severe		29 (29.9)	25 (28.1)	4 (50.0)	
Critical		7 (7.2)	6 (6.7)	1 (12.5)	
**Clinical characteristics**					
Total hospitalization (days)	–	17 (9–23)	17 (9–24)	10 (6–10)	**0.012**
Invasive ventilation	–	12 (12.4)	9 (10.1)	3 (37.5)	**0.024**
**B: Study cohort II**					
	**Missing data**	**All** **(*****n =*** **13)**	**Survivors** **(*****n =*** **9)**	**Non-survivors** **(*****n =*** **4)**	
**Parameter**	***n***	***n*** **(%)** **Median (IQR)**	***n*** **(%)** **Median (IQR)**	***n*** **(%)** **Median (IQR)**	***P*** -value^*^
**Sex**	1				> 0.999
Male		9 (69)	6 (67)	3 (75)	
Female		4 (31)	3 (33)	1 (25)	
**Age (years)**	1	75 (74–84)	73 (67–82)	81 (79–84)	0.328
**Comorbidities**					
Current smoker	1	1 (8.3)	1 (12.5)	0 (0.0)	0.460
Obesity (BMI > 25)	3	7 (70.0)	6 (100.0)	1 (25.0)	**0.011**
Diabetes type II	1	4 (33.3)	3 (37.5)	1 (25.0)	0.665
Hypertension	1	9 (75.0)	6 (75.0)	3 (75.0)	> 0.999
Cardiovascular disease (any)	1	6 (50.0)	3 (37.5)	3 (75.0)	0.221
Coronary heart disease	1	2 (16.7)	1 (12.5)	1 (25.0)	0.584
Chronic heart failure	1	2 (16.7)	1 (12.5)	1 (25.0)	0.584
Atrial fibrillation	1	3 (25.0)	2 (25.0)	1 (25.0)	> 0.999
Peripheral arterial disease	1	1 (8.3)	1 (12.5)	0 (0.0)	0.460
Chronic obstructive pulmonary disease	1	2 (16.7)	2 (25.0)	0 (0.0)	0.273
Asthma	2	0 (0.0)	0 (0.0)	0 (0.0)	
Hypo-/Hyperthyroidism	1	3 (25.0)	2 (25.0)	1 (25.0)	> 0.999
Chronic renal insufficiency	1	1 (8.3)	0 (0.0)	1 (25.0)	0.140
Chronic liver disease	1	1 (8.3)	1 (12.5)	0 (0.0)	0.460
Malignancy	1	4 (33.3)	3 (37.5)	1 (25.0)	0.665
**Medication (anti-platelet/anticoagulation)**					
Anti-platelet therapy	1	3 (25.0)	2 (25.0)	1 (25.0)	> 0.999
Anticoagulation therapy	1	12 (100.0)	8 (100.0)	4 (100.0)	
**COVID-19 classification at admission^†^**	2				0.449
Asymptomatic/mild		1 (9.1)	1 (12.5)	0 (0.0)	
Moderate		9 (81.8)	6 (75.0)	3 (100.0)	
Severe		1 (9.1)	1 (12.5)	0 (0.0)	
Critical		0 (0.0)	0 (0.0)	0 (0.0)	
**Clinical characteristics**					
Total hospitalization (days)	1	16 (12–19)	16 (14–18)	15 (10–19)	0.666
Invasive ventilation	1	0 (0.0)	0 (0.0)	0 (0.0)	

* *p < 0.05. Nominal variables were compared using the Chi-square test, metric variables were compared using T-test*.

† *COVID-19 classification according to the guidelines issued by the World Health Organization in mild (fever <38°C, no dyspnea, no pneumonia), moderate (fever, respiratory symptoms, pneumonia), severe (respiratory distress with respiratory rate ≥30 per minute, oxygen saturation <93% at rest) and critical (respiratory failure with requirement of mechanical ventilation, requirement of ICU)*.

*BMI: body mass index; IQR: interquartile range*.

*Statistically significant changes are highlighted in bold*.

**Table 2:** Laboratory findings at admission flow cytometric study cohort I **(A)** and proteomics cohort II **(B)**.

**A: Study cohort I**					
	**Missing data**	**All** **(*****n =*** **97)**	**Survivors** **(*****n =*** **89)**	**Non-survivors** **(*****n =*** **8)**	
**Parameter**	***n***	**Median (IQR)**	**Median (IQR)**	**Median (IQR)**	***P*** -value^*^
Hemoglobin (g/dL)	3	13.1 (12.1–14.6)	13.2 (12.3–14.7)	11.9 (11.1–13.3)	0.093
Red blood cell count (× 10^12^/L)	3	4.5 (4.1–5.0)	4.6 (4.1–5.0)	4.0 (3.5–4.4)	**0.043**
Platelet count (× 10^9^/L)	3	205 (140–226)	200 (137–226)	261 (183–283)	0.348
Leukocyte count (× 10^9^/L)	3	6.0 (4.0–7.2)	6.0 (3.9–7.2)	6.8 (5.0–7.4)	0.494
Lymphocyte count (× 10^9^/L)	7	1.0 (0.6–1.2)	1.0 (0.7–1.2)	0.8 (0.6–0.8)	0.148
Neutrophil count (× 10^9^/L)	7	4.7 (3.0-5.9)	4.6 (3.0–5.8)	5.5 (3.8–6.1)	0.492
Monocyte count (× 10^9^/L)	7	0.4 (0.2–0.5)	0.4 (0.2–0.4)	0.5 (0.3–0.6)	0.078
Eosinophil count (× 10^9^/L)	7	0.03 (0.00–0.03)	0.03 (0.00–0.03)	0.02 (0.01–0.03)	0.834
Basophil count (× 10^9^/L)	7	0.03 (0.01–0.03)	0.03 (0.01–0.04)	0.02 (0.01–0.02)	0.208
C–reactive protein (mg/L)	3	65.8 (31.5–82.6)	65.4 (29.2–82.6)	69.6 (35.6–88.7)	0.982
D–dimer (mg/dL)	15	0.8 (0.5–1.5)	0.8 (0.5–1.0)	1.7 (0.7–1.9)	0.219
Prothrombin time (%)	6	97.8 (89.7–109.3)	98.0 (89.7–110.8)	95.9 (91.6–102.5)	0.453
International normalized ratio	6	1.1 (1.0–1.1)	1.1 (1.0–1.1)	1.0 (1.0–1.1)	0.352
Activated partial thromboplastin time (s)	10	33.8 (29.4–37.1)	33.8 (29.2–37.0)	34.2 (30.3–37.9)	0.533
**B: Study cohort II**					
***Study cohort II***	**Missing data**	**All** **(*****n =*** **13)**	**Survivors** **(*****n =*** **9)**	**Non-survivors** **(*****n =*** **4)**	
**Parameter**	***n***	**Median (IQR)**	**Median (IQR)**	**Median (IQR)**	***P*** -value^*^
Hemoglobin (g/dL)	1	13.3 (12.2–14.4)	13.2 (12.9–14.2)	13.4 (12.2–14.5)	0.885
Red blood cell count (× 10^12^/L)	1	4.4 (4.1–4.7)	4.4 (4.3–4.6)	4.5 (4.0–4.9)	0.765
Platelet count (× 10^9^/L)	1	214 (184–238)	223 (201–238)	196 (178–200)	0.299
Leukocyte count (× 10^9^/L)	1	8.4 (4.9–10.6)	8.9 (5.6–10.6)	8.1 (4.4–10.5)	0.781
Lymphocyte count (× 10^9^/L)	4	0.9 (0.5–1.1)	1.0 (0.6–1.4)	0.6 (0.5–0.7)	0.331
Neutrophil count (× 10^9^/L)	4	6.7 (2.7–7.8)	7.7 (3.8–10.9)	4.7 (3.2–5.7)	0.352
Monocyte count (× 10^9^/L)	4	0.4 (0.4–0.5)	0.4 (0.4–0.5)	0.3 (0.2–0.3)	0.181
Eosinophil count (× 10^9^/L)	4	0.05 (0.01–0.06)	0.07 (0.02–0.08)	0.02 (0.02–0.03)	0.289
Basophil count (× 10^9^/L)	4	0.08 (0.05–0.10)	0.09 (0.07–0.11)	0.05 (0.04–0.05)	0.083
C–reactive protein (mg/L)	1	117.1 (76.9–154.5)	111.5 (63.1–168.0)	128.4 (123.7–148.1)	0.933
D–dimer (mg/dL)	5	1.3 (1.1–1.8)	1.1 (0.9–1.4)	1.8 (1.2–2.5)	0.762
Prothrombin time (%)	3	93.5 (79.1–106.7)	91.8 (79.1–104.1)	95.9 (86.7–106.5)	0.724
International normalized ratio	4	1.1 (1.0–1.2)	1.1 (1.0–1.2)	1.1 (1.0–1.1)	0.841
Activated partial thromboplastin time (s)	3	32.7 (30.2–35.8)	32.5 (29.9–35.8)	32.9 (30.8–33.4)	0.914

* *p < 0.05. Metric variables were compared using T- test or Mann-Whitney test; IQR, interquartile range*.

*Statistically significant changes are highlighted in bold*.

### A Drop in Basal Platelet Activation Level in Non-surviving COVID-19 Patients

In the early stages of severe COVID-19, increased basal platelet activation has been demonstrated (28, 29). We determined dynamic changes of basal platelet activation during hospitalization. For this purpose, we examined the platelets on study day 0 and after 4–5 days after inclusion in the study in patients who died with COVID-19 (*n* = 8) in comparison with survivors (*n* = 89). Basal platelet activation was determined by measuring the percentage of the activated integrin αIIbβ3 (CD41/CD61) complex on the membrane surface by flow cytometry. We focused on the glycoprotein αIIbβ3 rather than CD62P as activation marker since CD62P as activation marker is prone to time-dependent shedding. The activation-dependent upregulation of CD62P from the alpha granules, which is widely used as degranulation marker, can also be shed from the surface in the case of very strong platelet activation and thus become less again on the surface of platelets. Moreover, the quantification of the activation-dependent conformational change of the glycoprotein αIIbβ3 provides also the link between platelet activation and fibrinogen binding and thus platelet aggregation.

**Figure 1:**
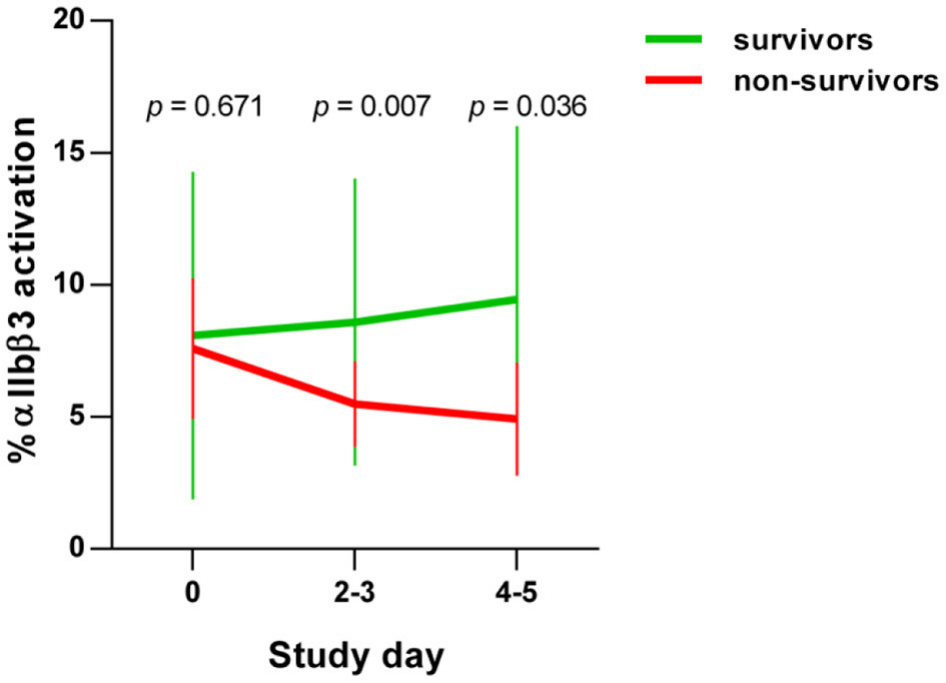
Basal platelet activation in COVID-19 patients. Activation of integrin αIIbβ3 complex on platelets from COVID-19 patients, detected by PAC-1 antibody binding. Basal platelet activation was specified as % binding of the FITC-labeled PAC-1 antibody and depicted as mean ± 95% confidence interval (CI).

On the day of study entry, day 0, no significant difference in integrin αIIbβ3 activation was observed between survivors and non-survivors among COVID-19 patients. At days 2–3 and days 4–5, however, a significant decrease in the activated integrin αIIbβ3 complex was detected in non-surviving COVID-19 patients (Figure 1). This apparent decrease in the basal platelet activation state in COVID-19 patients corresponds to a contradicting platelet phenotype, which, however, is often observed in diseases with an increasing incidence of thrombotic and fatal courses. For example exhausted platelets are described in patients with chronic obstructive pulmonary disease (42), sepsis (43) acute stroke (44), and cancer types with high risk of venous thromboembolism (45). Due to the continuous activation of the platelets in these conditions, exhaustion, or hypo-reactivity of the platelets is assumed. An alternative and non-mutually exclusive explanation is that activated platelets do not circulate but are rapidly removed from the circulation (46). More precise dynamic changes in the biochemical processes of such “hypo-reactive” platelets in the circulation are largely unknown in these different diseases with a high risk of thrombosis, as in our current COVID-19 patients.

### Outcome-Related Alterations in the Platelet Proteome of Patients With COVID-19 With Comparison to Healthy Controls

**Figure 2:**
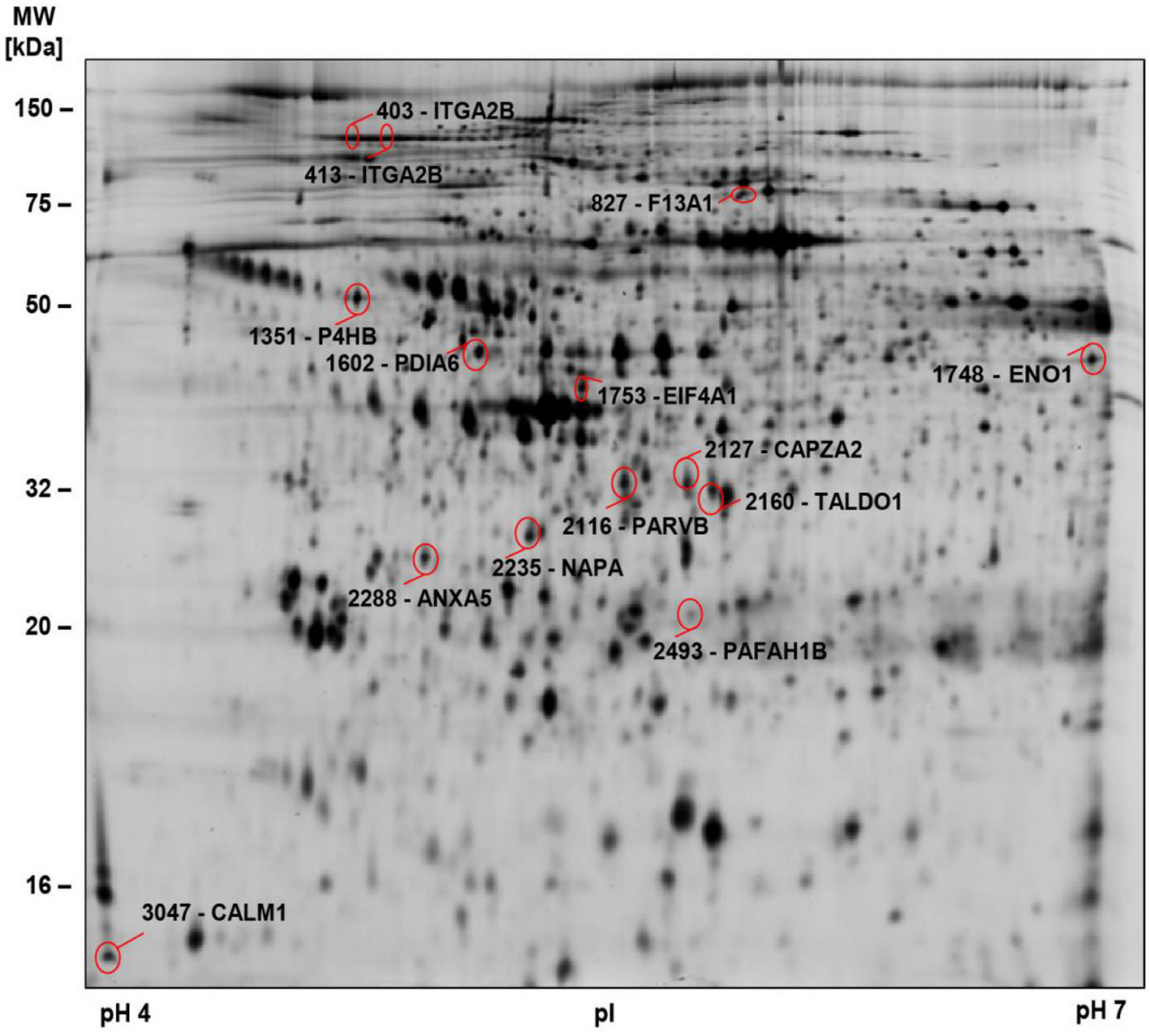
2D-DIGE-based proteome analysis of platelets from COVID-19 patients compared to controls. Representative 2D-DIGE image of protein spots with significant alterations in COVID-19 patients compared to controls (see Table 3). Platelet protein extracts were separated according to the isoelectric point (pI) in the pH 4–7 range and the molecular weight (MW). Protein spots identified by MS are circled and labeled with their corresponding gene name and spot numbers. Detailed descriptions of the highlighted proteins are listed in Table 3.

**Figure 3:**
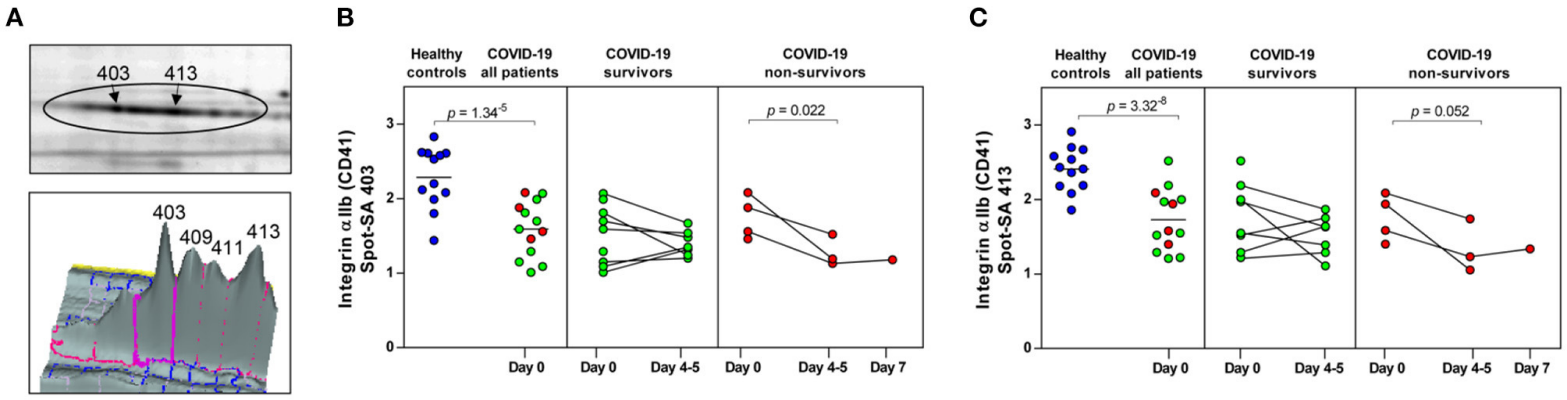
COVID-19 and mortality-dependent course of the abundance of integrin αIIb in platelets. **(A)** Illustration of the 2-D profile of the integrin αIIb (ITGA2B or CD41) spot chain from the 2D-DIGE analysis. **(B,C)** Protein levels of the ITGA2B proteoforms (spot 403 and 413). Scatter dot plot and time course of COVID-19 patients of ITGA2B standardized abundance (SA) of the platelet protein spots quantified by 2D-DIGE (healthy controls: *n* = 12; COVID-19 survivors: *n* = 9; COVID-19 non-survivors: *n* = 4). Protein levels were depicted as single values and mean. 2D-DIGE, two-dimensional differential in-gel electrophoresis.

To gain a deeper insight into the biochemical changes of platelets in COVID-19 and to determine differences between survivors and non-survivors, we examined the platelet proteomes of 9 surviving and 4 non-surviving COVID-19 patients and compared these with 12 healthy controls (Figure 2, Supplementary Figure 2; Table 1 study cohort II). Similar to basal αIIbβ3 activation, the platelet proteome of COVID-19 patients was determined on study day 0 and after 4–5 days using 2D-DIGE analysis in the pH range 4–7 (Figure 3). After applying selection criteria for the quality of the comparable protein spots (defined in the Materials and Methods section) on the 2D-DIGE gels, a total of 420 protein spots were included in the exploratory statistical data analysis.

**Figure 4:**
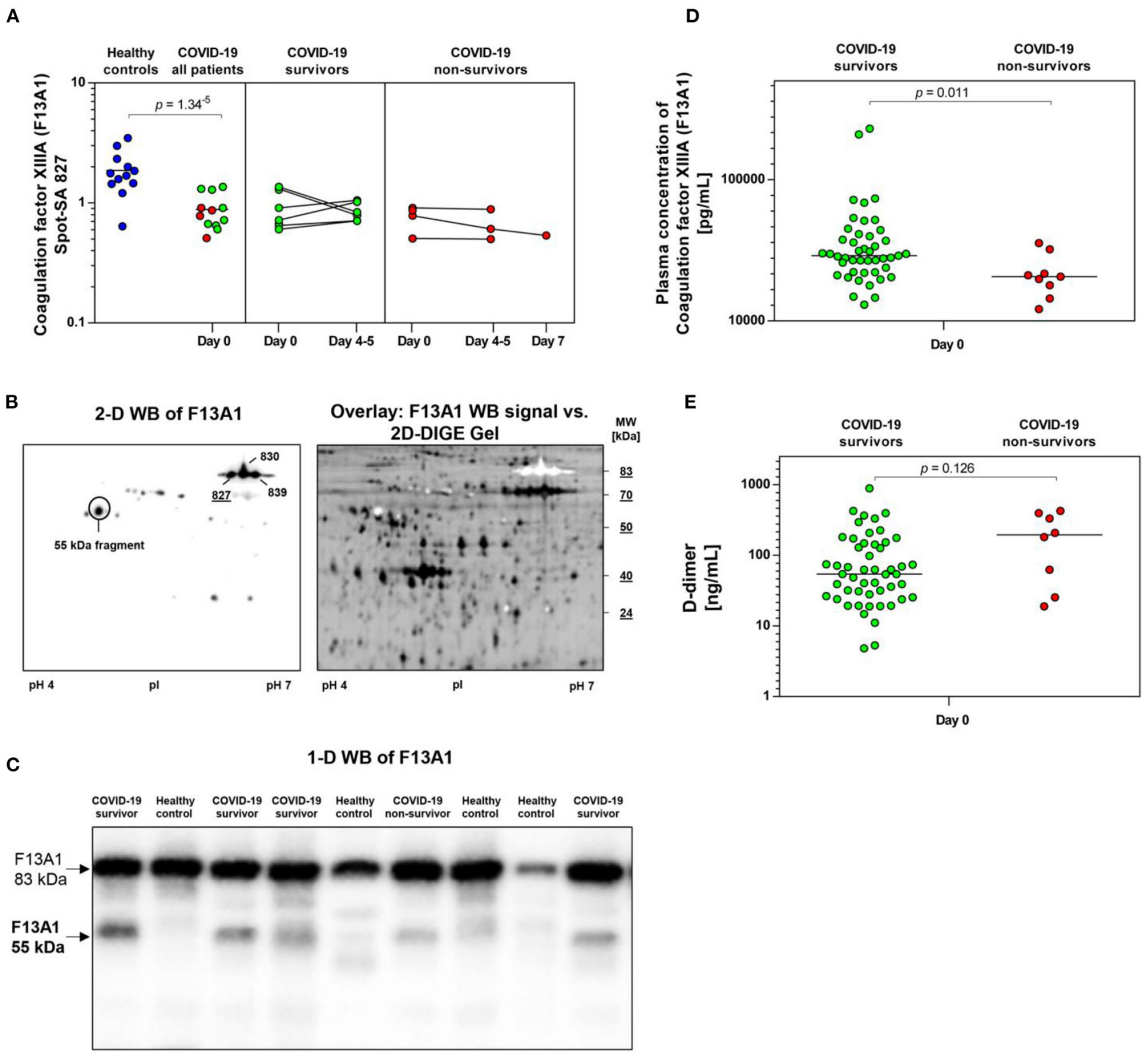
COVID-19-dependent course of the abundance of F13A1 in platelets. **(A)** Protein levels of F13A1 proteoform (spot 827). Scatter dot plot and time course of COVID-19 patients of F13A1 standardized platelet protein spot abundance quantified by 2D-DIGE. (healthy controls: *n* = 12; COVID-19 survivors: *n* = 9; COVID-19 non-survivors: *n* = 4). **(B)** Platelet proteins were separated according to their molecular weight (MW) and isoelectric point (pI). 2-D western blot (WB) image of platelet F13A1 probed with monoclonal anti-F13A1 antibody (left). Cy2-labeled protein was applied to IEF on a 24 cm pH 4–7 IPG-strip. Overlay of whole protein (black) and F13A1 signal (white) (right). Overlay of 2D-DIGE gel vs. F13A1 WB-signal, obtained through the Online Image Editor (https://www.online-image-editor.com). **(C)** Representative 1-D WB image of F13A1 in platelet proteins from COVID-19 survivors (*n* = 4), COVID-19 non-survivors (*n* = 1) and healthy controls (*n* = 4). The anti-F13A1 antibody detects two protein bands with molecular weight of 83 and 55 kDa. **(D)** Plasma F13A1 concentration at day 0 (COVID-19 survivors: *n* = 45; COVID-19 non-survivors: *n* = 8). **(E)** Plasma levels of D-dimer at day 0 (COVID-19 survivors: *n* = 45; COVID-19 non-survivors: *n* = 8). Protein levels of F13A1 and D-dimer were depicted as single values and mean. 2D-DIGE, two-dimensional differential in-gel electrophoresis; kDa, kilodalton.

**Figure 5:**
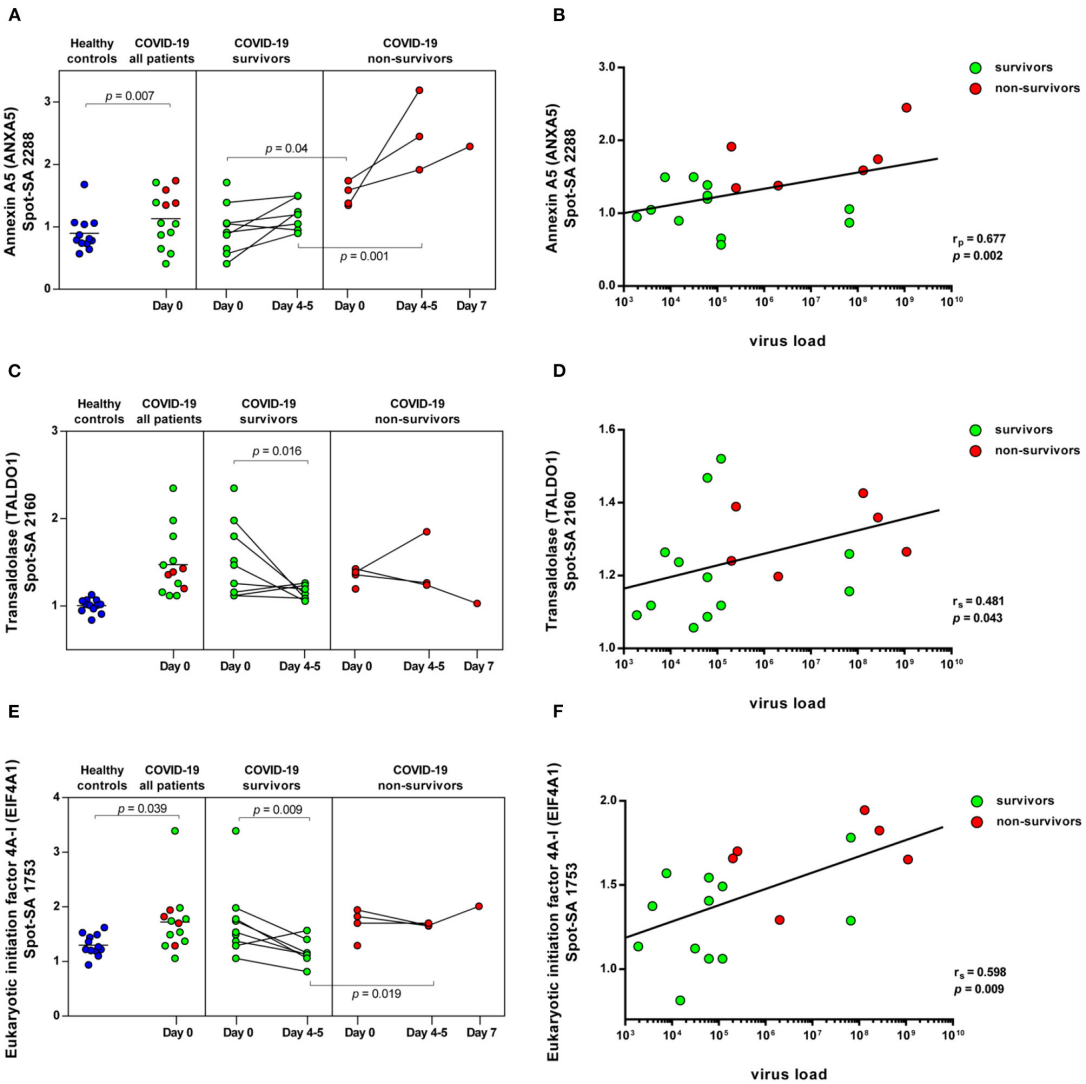
COVID-19-dependent course of the abundance of ANXA5, TALDO1 and EIF4A1 in platelets. Scatter dot plot and time course of ANXA5, TALDO1, and EIF4A1 standardized platelet protein spot abundance in COVID-19 patients quantified by 2D-DIGE (healthy controls: *n* = 12; COVID-19 survivors: *n* = 9; COVID-19 non-survivors: *n* = 4) and their correlation with nasopharyngeal virus load. **(A)** Protein levels of ANXA5 (spot 2288) and **(B)** scatter dot plot correlation analysis (Pearson's Rank correlation coefficient) of virus load and 2D-DIGE ANXA5 levels. **(C)** Protein levels of TALDO1 (spot 2160) and **(D)** scatter dot plot correlation analysis (Spearman's Rank correlation coefficient) of virus load and 2D-DIGE TALDO1 levels. **(E)** Protein levels of EIF4A1 (spot 1753) and **(F)** scatter dot plot correlation analysis (Spearman's Rank correlation coefficient) of virus load and 2D-DIGE EIF4A1 levels. 2D-DIGE, two-dimensional differential in-gel electrophoresis.

**Figure 6:**
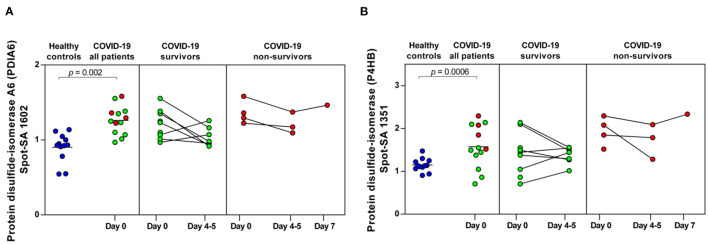
COVID-19-dependent course of the abundance of PDIA6 and P4HB in platelets. **(A)** Protein levels of PDIA6 (spot 1602) and **(B)** of P4HB (spot 1351). Scatter dot plot and time course of COVID-19 patients of PDIA6 and P4HB standardized platelet protein spot abundance quantified by 2D-DIGE (healthy controls: *n* = 12; COVID-19 survivors: *n* = 9; COVID-19 non-survivors: *n* = 4). 2D-DIGE, two-dimensional differential in-gel electrophoresis.

Significant alterations in platelet protein levels between COVID-19 survivors and non-survivors at day 0 and day 4–5 and relative to healthy controls were filtered out by one-way ANOVA, revealing 44 significantly changed protein spots. To determine COVID-19-related platelet protein changes, a planned contrast analysis was carried out between all COVID-19 patients on day 0 and healthy controls. These hypothesis-directed statistical tests limited the number to 14 significantly altered COVID-19-related platelet proteins shown in Figure 2 and given in Table 3. The following Figures (Figures 3B,C, 4A, 5A,C,E, 6A,B) show these platelet protein alterations for the individual patients in comparison to healthy controls. The consistency of these COVID-19-dependent protein courses is also documented in a non-survivor on day 7.

**Table 3:** 2D-DIGE-identified proteome alterations in platelets from COVID-19 patients compared to healthy controls.

						**All COVID-19 patients/Healthy controls**			**Non-survivors/Survivors**				**Day 4–5/Day 0**			
							**Day 0**		**Day 0**		**Day 4-5**		**Survivors**		**Non-survivors**	
**Spot number**	**Protein name**	**Uni-Prot number**	**Gene name**	**MW [kDa]**	**pI**	***P*-value of One-way ANOVA**	**FC**	***P*-value**	**FC**	***P*-value**	**FC**	***P*-value**	**FC**	***P*-value**	**FC**	***P*-value**
413	Integrin αIIb	P08514	ITGA2B	113	4.80	0.0001	0.72	3.32E-08	1.02	0.417	0.88	0.367	0.89	0.411	0.88	0.052
403	Integrin αIIb	P08514	ITGA2B	113	4.50	0.0005	0.67	1.34E-05	1.15	0.213	0.92	0.350	0.92	0.694	0.92	0.022
2160	Transaldolase	P37837	TALDO1	37	6.36	0.0002	1.47	2.78E-06	0.88	0.345	1.26	0.068	0.75	0.016	1.26	0.733
2288	Annexin A5	P08758	ANXA5	35	4.93	0.0005	1.26	0.007429	1.58	0.040	2.12	0.001	1.24	0.055	2.12	0.078
1602	Protein disulfide-isomerase A6	Q15084	PDIA6	48	4.95	0.0006	1.40	0.001516	1.12	0.343	1.17	0.095	0.86	0.067	1.17	0.333
827	Coagulationfactor XIIIA	P00488	F13A1	83	5.65	0.0007	0.58	0.000157	0.61	0.220	0.78	0.122	0.68	0.217	0.78	0.777
2493	Platelet-activating factor acetylhydrolase IB subunit α2	P68402	PAFAH1B2	25	5.57	0.0012	1.79	0.000225	0.86	0.864	1.96	0.029	0.60	0.028	1.96	0.256
2116	β-parvin	Q9HBI1	PARVB	35	6.25	0.0029	1.34	0.000139	0.99	0.565	0.98	0.484	0.91	0.946	0.98	0.332
1748	α-enolase	P06733	ENO1	47	7.01	0.0036	0.65	0.000281	1.02	0.779	1.09	0.240	0.99	0.751	1.09	0.937
2235	α-soluble NSF attachment protein (SNAP-α)	P54920	NAPA	33	5.23	0.0055	1.41	0.001080	0.98	0.750	1.01	0.918	0.81	0.316	1.01	0.359
1753	Eukaryotic initiation factor 4A-I	P60842	EIF4A1	44	5.32	0.0055	1.33	0.038561	0.97	0.191	1.41	0.019	0.74	0.009	1.41	0.481
2127	F-actin-capping protein subunit α-2	P47755	CAPZA2	33	5.57	0.0057	1.56	0.000448	0.77	0.446	1.33	0.326	0.74	0.088	1.33	0.510
3047	Calmodulin	P0DP23	CALM1	16	4.09	0.0078	1.70	0.105430	1.25	0.489	0.95	0.299	0.62	0.083	0.95	0.771
1351	Protein disulfide-isomerase (P4HB)	P07237	PDIA1	57	4.76	0.0121	1.37	0.000595	1.37	0.125	1.25	0.124	0.97	0.581	1.25	0.271

*The p-values (p ≤ 0.01) of the one-way ANOVA indicate the variance of the respective proteoforms between the five groups of surviving and non-surviving COVID-19 patients on day 0 and day 4–5 and healthy controls. COVID-19-related platelet protein changes are characterized by planned contrast analysis (p ≤ 0.05) between all patients with COVID-19 (n = 13) from day 0 and healthy controls (n = 12) and the average fold-change (FC). The evaluation of outcome-related changes of these COVID-19-related proteins are calculated by planned contrast analysis from the survivors and non-survivors on day 0 and day 4–5. The planned contrast analysis (p ≤ 0.05) indicates proteins, which are significantly changed dependent from the outcome on day 0 and day 4–5 as well as between day 0 and day 4–5. All these calculations are carried out with the values of the standardized protein abundance, quantified with the 2D-DIGE system*.

*2D-DIGE, two-dimensional differential in-gel electrophoresis; MW, molecular weight; pI, isoelectric point; FC, fold change*.

Unexpectedly, when comparing patients to healthy controls this unbiased proteomics analysis revealed the strongest COVID-19-related influence on the total amount of integrin αIIb (ITGA2B; CD41; spot 413: FC = 0.72, *p* = 3.32^−8^) part of the platelet integrin αIIbβ3 complex, which was highly significantly decreased (Figure 2, Table 3).

ITGA2B is a multiply glycosylated protein in platelets and is therefore visible in the 2-D proteome map as a protein chain with 10 protein spots (proteoforms) with different isoelectric points (pI), which reflect different degrees of glycosylation. The very strict qualitative selection criteria used included only two of these ITGA2B proteoforms (Figure 3A) in the statistical analyses, spot 403 (Figure 3B) and 413 (Figure 3C), which represent the two most abundant forms of this integrin (Figure 3A). The other ITGA2B proteoforms were generally similarly regulated in COVID-19 patients, but data did not reach a reproducibility of 95%.

An additional planned contrast analysis of the time course of ITGA2B levels in platelets further showed a significant decrease of ITGA2B over time in non-surviving COVID-19 patients (Figures 3B,C), which fits to the decrease in the activated integrin-αIIbβ3 complex observed in non-survivors by flow cytometry (Figure 1).

A STRING protein network analysis (Supplementary Figure 4) showed that ITGA2B together with coagulation factor XIIIA (F13A1), annexin A5 (ANXA5) and calmodulin (CALM1)—all significantly altered in COVID-19 patients relative to healthy donors (Table 3)—significantly enriched the biological process “platelet degranulation” (*p* = 0.0243), thereby also confirming the recent bioinformatic meta-assessment results of several previous clinical proteomics studies of COVID-19 patients demonstrating increased platelet degranulation (47). F13A1 is represented in our 2D-DIGE platelet protein map by three 83 kDa proteoforms (pI 5.85 to pI 6.05), though only one of them was significantly changed in COVID-19 patients relative to healthy controls (Table 3, Figures 4A,B). This proteoform with the pI 5.65 was significantly decreased in COVID-19 patients (FC = 0.58; *p* = 0.0002). As the last factor in the coagulation cascade, this transglutaminase catalysis the irreversible cross-linking of fibrin and thus ensures the formation of a stable thrombus. In a previous platelet proteomics study of patients with lung cancer, we could show an accelerated inactivation of F13A1 via an elevated amount of a 55 kDa fragment of F13A1 (36). Also in the current study, the COVID-19-dependent, significant reduction of the F13A1 proteoform with pI 5.65 could be caused by its increased breakdown. However, due to the rigorous access-restrictions of personnel to COVID-19 samples, optimal sample preparation was not possible during bio banking work and platelets were only washed once. This limitation led to a higher level of plasma proteins in the platelet proteome than in our previous studies. Consequently, we could not detect the 55 kDa inactivation product of F13A1, as its spot area was overlaid by spots of the plasma protein SERPINA1. Nevertheless, a 2-D Western blot analysis of the internal standard sample (pool of all proteomics study samples) detected this particular 55 kDa F13A1 fragment immunologically, demonstrating F13A1 degradation (Figure 4B). A 1-D Western blot analysis further showed that this 55 kDa F13A1 fragment was clearly detectable in platelets of COVID-19 patients regardless of the outcome, but not in the healthy controls (Figure 4C). An increased consumption of F13A1 linked to a decrease in the F13A1 concentration in the plasma has already been described earlier in various thrombotic diseases, such as acute deep vein thrombosis (48). Similarly, the F13A1 concentration in the plasma of COVID-19 non-survivors was significantly reduced compared to the surviving COVID-19 patients (FC = 0.71; *p* = 0.011) (Figure 4D), even though the total F13A1 amount in platelets (sum of all 83 kDa proteoform spots) was not found to be different (data not shown). Statistically, this decrease in F13A1 in plasma was higher significant than the increase in the degradation product of cross-linked fibrin, D-dimer (FC = 1.81; *p* = 0.126, Figure 4E).

ANXA5, the major annexin in human platelets, was significantly increased in COVID-19 patients compared to healthy controls (FC = 1.26; *p* = 0.007). This platelet protein showed the highest association with mortality among the identified significantly altered proteins (Table 3) with a significantly increased amount in the deceased compared to surviving COVID-19 patients (FC = 1.58; *p* = 0.040) on day 0 and even more so on day 4–5 (FC = 2.12; *p* = 0.001; Figure 5A). It has been shown that ANXA5 of an influenza virus-infected cell can be incorporated into the virus particle (49). In the current study, a correlation of ANXA5 with the SARS-CoV-2 exposure of COVID-19 patients was found (r_p_ = 0.677; *p* = 0.002; *n* = 18; Figure 5B). The virus load was quantified by means of a nasopharyngeal swab (Supplementary Figure 5). For the correlations of the viral load with the respective amount of the platelet protein, the different points in time were combined.

Transaldolase (TALDO1), an enzyme of the carbohydrate metabolism, was also significantly increased in COVID-19 patients compared to the healthy controls, however levels declined over time in survivors almost to levels of healthy controls (Figure 5C). TALDO1 is a part and modulator of the pentose phosphate pathway which can also supply ribonucleotides for virus replication. Therefore, TALDO1 could be a potential drug target for antiviral interventions. In line with these facts, the TALDO1 levels of the platelets correlated with the nasopharyngeal virus load of the COVID-19 patients (r_S_ = 0.481; *p* = 0.043; *n* = 18; Figure 5D).

With a significant increase in EIF4A1 in platelets of COVID-19 patients, we identified another protein that may be directly related to viral RNA translation (50, 51). The association of mortality with EIF4A1 was similar to that of TALDO1, with a significant decrease (FC = 0.74; *p* = 0.009) in survivors between days 0 and days 4–5. At the same time, levels in non-survivors remained stable, resulting in significantly increased levels of EIF4A1 in the non-surviving COVID-19 patients compared to the survivors on days 4–5 (FC = 1.41; *p* = 0.019; Figure 5E). Furthermore, we also found a significant correlation between the amount of EIF4A1 in platelets and nasopharyngeal viral load of COVID-19 patients (r_S_ = 0.598; *p* = 0.009; *n* = 18; Figure 5F).

Finally, two members of the protein disulfide isomerase (PDI) family, PDIA6 and P4HB, which are critically responsible for thrombus formation (52), were significantly increased in COVID-19 patients compared to healthy controls (PDIA6 spot 1602: FC = 1.40; *p* = 0.002 and P4HB spot 1351: FC = 1.37; *p* = 0.0006). Both thiol isomerases showed a trend toward higher levels in the non-surviving COVID-19 patients, although the results were not significant (Figures 6A,B).

## Discussion

COVID-19-associated advanced severe inflammation in the lungs is often seen associated with massive viral invasion and widespread severe thrombotic microangiopathy. The SARS-CoV-2 RNA is also detectable in platelets of COVID-19 patients (31) and the virus directly causes a hyper-activation of the platelets (26). Platelets of severely ill COVID-19 patients were shown to be more activated compared to healthy and mild courses (28). In this study, we monitored COVID-19 patients over a period of 5 days and found that (1) basal integrin αIIbβ3 activation in platelets of non-surviving COVID-19 patients was decreasing compared to survivors. In addition, using an unbiased platelet proteome analysis, we found that (2) the total amount of one part of this integrin complex, ITGA2B, was decreased in all COVID-19 patients compared to healthy controls, and in non-survivors the decrease was even stronger after 4–5 days. COVID-19 dependent changes in the fibrin-crosslinking system were demonstrated (3) by an increased consumption of intact F13A1 in platelets, which was even more pronounced in the plasma of non-surviving COVID-19 patients. The abundance of (4) ANXA5 was significant higher in non-surviving COVID-19 patients on day 0 compared to survivors with an even higher increase on days 4–5. This phospholipid-binding has already been previously characterized as an autoantigen of the antiphospholipid syndrome (APS) of COVID-19. Finally, two mortality-dependent changes in the platelet proteome were identified in COVID-19 patients, which may be directly related (5) to virus replication. On the one hand, we found an increased level of the EIF4A1, which also enables viral RNA translation, on the other hand we observed increased amounts of the enzyme transaldolase, which supplies ribonucleotides for virus replication.

Our initial finding was the decrease in basal platelet activation in non-surviving COVID-19 patients within 4–5 day observation period, which was detected by the activated integrin-αIIbβ3 complex. At the first glance, this reduction in platelet activation status in non-surviving COVID-19 patients contradicts previous study results that showed elevated platelet activation in patients with severe vs. mild COVID-19 disease (28, 29). However, platelet activation status has not been monitored over time during COVID-19. With a subsequent unbiased analysis of the proteome, we took a closer look at the dynamic changes of the platelet phenotype and the association with outcome of COVID-19 and also included a cohort of healthy controls. Unexpectedly, the proteome analysis showed the strongest change with a highly significant reduction in the total amount of ITGA2B in platelets from COVID-19 patients compared to healthy controls and—similar to αIIbβ3 activation data—an even stronger decrease in non-surviving patients compared to surviving COVID-19 patients. Of note, in diseases with a high risk of thrombosis, such as lung cancer (36) and lupus anticoagulants (53), we have previously observed a decrease in the total ITGA2B level in the platelet proteome, but not to this extent, underlining the magnitude of thrombotic dysregulation in COVID-19.

This depletion of ITGA2B in platelets during this prothrombotic disease may be caused by the continuous hyper-activation of platelets leading to persistent degranulation and release of platelet extracellular vesicles. These membrane shed vesicles contain fairly high levels of ITGA2B (54) and their continuous release can lead to a decrease in the absolute amount of ITGA2B in the whole platelets as well as on their surface. In fact, elevated platelet extracellular vesicles concentrations were detected in the plasma of COVID-19 patients (31). Thus, the drop of the activated αIIbβ3 complex on platelets of non-surviving COVID-19 patients, observed in the current study, may be attributed to a generally declining amount of total ITGA2B in their platelets due to hyper-activation. Strikingly, it has already been shown that platelets from COVID-19 patients, activated via the GPVI receptor (30, 32) show a reduced activation of the integrin-αIIbβ3 complex in comparison to controls. To establish a possible link to our results of diminished ITGA2B in COVID-19, it should be noted that the activation of the integrin αIIbβ3 complex via its altered conformation is quantified by the binding of the antibody PAC-1. Thus, in the case of reduced total ITGA2B amount in the platelets of COVID-19 patients, less PAC-1 binding signal may be detected compared to healthy controls, even during an increased activated state of the integrin-αIIbβ3 complex, and thus hypo-reactivity of the platelets may be concluded.

With the detection of a reduced abundance of an 83 kDa spot of the coagulation factor F13A1 out of a total of three 2D-DIGE -measured proteoforms in the platelet proteome of COVID-19 patients, we postulated an altered regulation of this fibrin-stabilizing enzyme. This last zymogen in the coagulation cascade can be activated by thrombin and also inactivated via further enzymatic cleavage by thrombin (55) or plasmin (56), with a resulting 55 kDa degradation product. The shift of F13A1 proteoforms from pI 6.05 to pI 5.85 is not related to the cleavage by these enzymes but, can be caused by the activation of the catalytic center via acetylation (57). However, we were unable to find these acetylations using MS analysis. Nevertheless, we could previously show that the COVID-19-related 83 kDa proteoform with pI 5.85 has the strongest correlation with the enzymatic activity of F13A1 in platelets, while the most alkaline F13A1 proteoform with the pI 6.05 is the inactive one (36). In addition, in our previous study we identified an accelerated processing of F13A1 in the platelet proteome in patients with lung cancer, which we recognized by an increased amount of its 55 kDa inactivation product. This F13A1 breakdown product could be detected immunologically only in COVID-19 patients with a 1-D Western blot. With simultaneous detection of a reduced level of an enzymatically active proteoform and an increased level of the 55 kDa inactivation product in the platelets of COVID-19 patients, it can be concluded that there is an increased consumption of F13A1 with a slightly stronger trend in non-survivors. Even more pronounced, a significantly lower concentration of F13A1 in the plasma of non-surviving COVID-19 patients compared to that of survivors additionally points also here to an accelerated consumption of F13A1. Interestingly, it has already been shown that the activity of F13A1 in the plasma of COVID-19 patients is strongly reduced compared to healthy controls and this decrease in F13A1 activity is more pronounced in patients admitted to a high-care facility than in patients admitted to general wards (58). The underlying mechanism behind this acquired plasma F13A1 deficiency in COVID-19 patients and other conditions with thrombotic complications (48, 59–61) is uncertain, but a consumptive mechanism has been suggested (62, 63). Our results of the altered F13A1 processing thus show a functionally explanatory mechanism for the increased consumption of F13A1 and an overarching pathological change in the fibrin stabilization system of platelets and in the plasma of COVID-19 patients.

Another change in platelet proteome that may be highly relevant for the pathogenesis of COVID-19 is the increasing amount of ANXA5 in the platelets of COVID-19 patients, which was significantly higher in non-survivors. ANXA5 belongs to a family of Ca^2+^ -dependent phospholipid-binding proteins and has strong anticoagulant and anti-apoptotic effects, which might theoretically counteract the prothrombotic effects of a SARS-CoV-2 infection. ANXA5 is pathologically associated with APS via the occurrence of anti-ANXA5 autoantibodies (64). These autoantibodies are described to neutralize the anticoagulant effect of ANXA5 derived from endothelium and can thus increase the risk of thrombosis in APS (65). In previous studies a considerable proportion (50–75%) of hospitalized COVID-19 patients has been diagnosed with APS (66–68). Interestingly, in the plasma of COVID-19 patients anti-ANXA5 autoantibodies were found more frequently than the usual antibodies mediating APS, (69). In a wider context, it is noteworthy that in systemic lupus erythematosus increased concentration of anti-ANXA5 antibodies was accompanied by an increased concentration of ANXA5 in the plasma. These increased plasma levels of ANXA5 correlated with corresponding platelet concentrations, which suggests that the ANXA5 found in the plasma, originates from the platelets (70). Overall these observations implicate, that the COVID-19-related increased levels of ANXA5 in platelets may also lead to increased concentrations of anti-ANXA5 antibodies and thus to COVID-19-related APS. In support of this hypothesis, it was also found that the level of autoantibodies against annexin A2, a protein important for fibrinolysis and the protection of lung tissue, predicts mortality in hospitalized COVID-19 patients (71). However, no association with mortality for autoantibodies against ANXA5 was found in this study (71). Notably, the presented results on elevated platelet ANXA5 levels in fatal COVID-19 courses should also be relevant to an ongoing study (NCT04748757), in which patients with severe COVID-19 courses are infused with recombinant ANXA5 to counteract inflammation and thrombosis.

SARS-CoV-2 can also directly enter into platelets, as platelets express angiotensin-converting enzyme 2 (ACE2), a host cell receptor for SARS-CoV-2, and transmembrane protease serine subtype 2 (TMPRSS2), a serine protease for spike protein priming. SARS-CoV-2-RNA has also been detected in platelets from COVID-19 patients (31). SARS-CoV-2 and its spike protein directly induce platelet activation (26) and can therefore also be directly responsible for the prothrombotic state of COVID-19.

The correlations of the COVID-19-dependent platelet proteins EIF4A1 and TALDO1 with the viral load of the patients also suggest a direct replication of the virus in platelets. Both platelet proteins are elevated in the COVID-19 patients on day 0 and then decrease after 4–5 days in the survivors, similar to their virus load, while all of them remain elevated in the non-survivors. In fact, platelets have previously been shown to replicate single-stranded RNA and produce viral protein from dengue virus and produce thereby infectious virus (72).

A SARS-CoV-2 protein interaction map recently identified the host's translational machinery as the primary target for blocking SARS-CoV-2 replication by interfering with one of the two main candidates, being EIF4A1 (73). The virus regulates the host processes involved in protein synthesis, such as the control of translation factors EIF4A. The helicase EIF4A is part of the cellular EIF4F translation initiation complex which is required for mRNA binding to the ribosome. In line, inhibitors of this factor such as Zotatifin and Rocaglate inhibit the EIF4A-dependent mRNA translation initiation, which leads to greatly reduced viral RNA translation in infected cells, including SARS-CoV-2 (51).

Thus, our results thus provide first evidence that a translational machinery in platelets are in fact accessible for SARS-CoV-2 replication. Likewise, the increased amount of the enzyme TALDO1 can be linked to increased activity of the pentose phosphate pathway, which supplies the virus with ribonucleotides, essential building blocks for its replication. In addition, an unbiased proteome analysis has previously demonstrated that the infection of Caco-2 cells with SARS-CoV-2 increases the expression of TALDO 1 (74).

In summary, similar to previously investigated prothrombotic conditions such as lupus anticoagulants and lung cancer, we found significantly increased levels of the two thrombosis-promoting protein disulfide isomerases P4HB and PDIA6 and a reduced total amount of ITGA2B in the platelets of COVID-19 patients. The F13A1 degradation was modulated in a similar way as in lung cancer. However, the significant changes regarding increased levels of ANXA5, EIF4A1 and TALDO1 are so far unique for the platelet proteome of COVID-19 patients.

Overall, our study has both limitations and strengths. From a statistical point of view, our study is exploratory, including a relatively small number of patients. Healthy controls used for proteomics analysis were not exactly matched to patient age and gender. Due to the limited sample size, it was not possible to carry out further statistical subgroup analyses of mild and severe COVID-19 courses in order to comprehensively assess the influence of the various degrees of severity of COVID-19 on the platelet proteome. Moreover, patients with pneumonia without SARS-CoV-2 infection and also SARS-CoV-2 infected patients without pneumonia would also be very important controls to find out which platelet proteome changes are specific and lethal for COVID-19. One of the strengths of our study is the repeated investigation of the platelet activation status as well as their proteome over a period of 4–5 days and their connection with mortality. The continuous mortality-dependent change in the platelet activation status during 4–5 days as well as the consistent course of many of the newly characterized COVID-19-dependent platelet protein changes underline their pathological relevance.

It is very important to note that the results of the current proteome analysis certainly did not capture all changes in the platelet proteome in COVID-19 patients. Due to the small number of cases, a very strict quality selection was carried out for the protein candidates included in the statistical proteome analysis. Therefore, some COVID-19-dependent protein changes in the platelets were probably not recorded. The selection of the proteome analysis method using the 2D-DIGE technology in the pH range 4–7 does not include the entire pH value 3–10 of the possible protein candidates. The 2-D analysis also has the disadvantage that it cannot detect lower concentrated proteins in biological samples and is therefore not as sensitive as LC-MS-based proteome analyses. A major advantage of the 2-D analysis, however, is that the biological samples do not have to be digested into peptides as with the shotgun proteomics analysis and thus intact proteins and their associated posttranslational modifications (PTMs) are directly analyzed qualitatively and quantitatively by 2-D (and 2D-DIGE). In fact, COVID-19 dependent reduction of only one (pI 5.85) out of three F13A1 proteoforms as well as changes of its 55 kDa inactivation product would have been undetectable by shotgun proteome analysis.

In any case, the identified COVID-19-dependent changes in platelets need to be validated in larger patient cohorts and potential functional relationships must be investigated as well as detailed *in vitro* studies on a possible direct replication of SARS-CoV-2 in platelets. Nevertheless, the platelet protein alterations detected in the current study, such as the increased concentration of ANXA5, seem important for the pathology of COVID-19 and should therefore be made available to the public as soon as possible.

Taken together, monitoring of the platelet phenotype of COVID-19 patients over a period of 4–5 days showed that the integrin αIIbβ3-based platelet activation status declined in non-survivors compared to survivors. The subsequent platelet proteome analysis provided the first evidence that this detection of a reduction in the activated integrin-αIIbβ3 complex was accompanied by a decrease in the total amount of one integrin component, ITGA2B. The current results suggest that in COVID-19 patients, continuous “degranulation” and the release of platelet microvesicles lead to features of “platelet exhaustion,” which is most likely caused by persistent platelet hyper-activation. With an increased consumption of F13A1 in platelets, which was even more pronounced in the plasma of the non-survivors, a strong change in the irreversible fibrin-crosslinking system occurred in fatal COVID-19 courses. In addition, platelets from non-survivors showed specific changes in proteins during this observational period that are closely related to the autoimmune response of APS and the replication of SARS-CoV-2. Thus, the results of the current proteomics study suggest that SARS-CoV-2 replication can also take place directly in the platelets and can therefore directly and specifically activate pathways of primary and secondary hemostasis. Accordingly, the acquired data state a central role of platelets not only in thromboinflammation during COVID-19 but also in the viral replication of SARS-CoV-2, thereby covering several main mechanisms in this disease. The implications of this study can be critical to a deeper understanding of the pathology and pathogenesis of COVID-19.

## Data Availability Statement

The raw data supporting the conclusions of this article will be made available by the authors, without undue reservation.

## Ethics Statement

The studies involving human participants were reviewed and approved by Ethics Committee of the Medical University of Vienna. The patients/participants provided their written informed consent to participate in this study.

## Author Contributions

HE, WS, BJ, AA, and MZ contributed to conception and design of the study. JS, EP, MT, CS, TS, MK, and AZ treated and recruited the COVID-19 patients. WS, AS, AP, and DP made the preparation of the samples and organized the database. HE and WS made the analysis of the patient samples. J-WY made MS analysis. MZ performed the statistical analysis. MZ and HE wrote the first draft of the manuscript. WS, HE, AA, DP, and J-WY wrote sections of the manuscript. All authors contributed to manuscript revision, read, and approved the submitted version.

## Funding

Austrian Federal Ministry of Education, Science and Research, the Medical-Scientific Fund of the Mayor of Vienna (COVID024) and the Austrian Science Fund (P-34783, P-32064; SFB-54P04).

## Conflict of Interest

The authors declare that the research was conducted in the absence of any commercial or financial relationships that could be construed as a potential conflict of interest.

## Publisher's Note

All claims expressed in this article are solely those of the authors and do not necessarily represent those of their affiliated organizations, or those of the publisher, the editors and the reviewers. Any product that may be evaluated in this article, or claim that may be made by its manufacturer, is not guaranteed or endorsed by the publisher.
