# Supplementary material for: Platelet Phenotype Analysis of COVID-19 Patients Reveals Progressive Changes in the Activation of Integrin αIIbβ3, F13A1, the SARS-CoV-2 Target EIF4A1 and Annexin A5
Source: Front Cardiovasc Med. 2021 Nov 11;8:779073. doi: 10.3389/fcvm.2021.779073 (PMC8632253; doi:10.3389/fcvm.2021.779073)
Supplement: Supplementary file 1 [file Data_Sheet_1.docx]

Supplementary Material

|  | **Healthy controls**  **(*n* = 12)** |
| --- | --- |
| **Parameter** | ***n* (%)**  **Median (IQR)** |
| **Sex** |  |
| Male | 7 (58.3) |
| Female | 5 (41.7) |
| **Age (years)** | 61 (44-63) |

*IQR: interquartile range.*

**Supplementary Table 1. Healthy controls demographics (Proteomics study cohort II).**


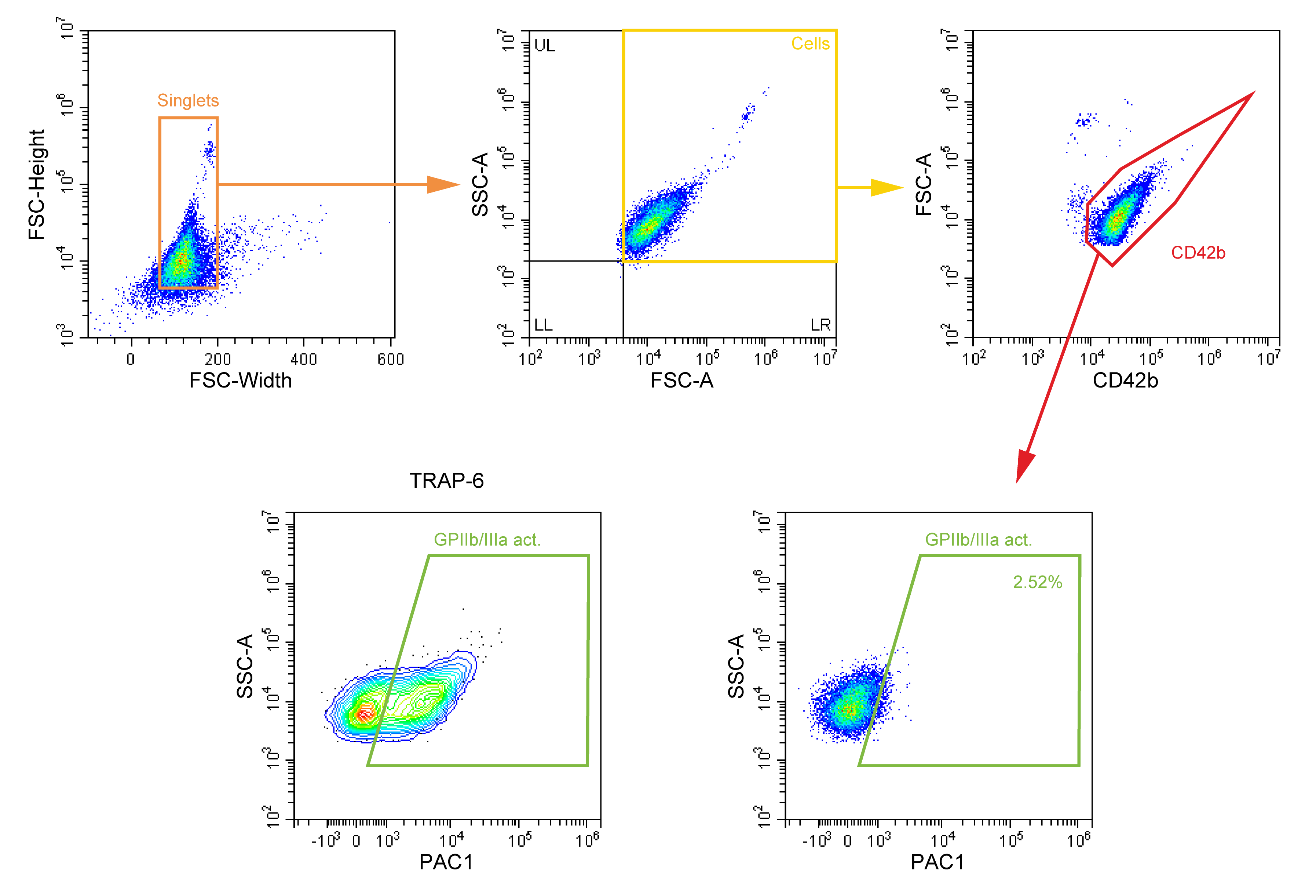


**Supplement Figure 1: Gating strategy in flow cytometry for GPIIb/IIIa quantification:** Singlet events were identified based on FSC-height and FSC-width and analysed for size using an FSC / SSC plot to identify intact cells. Platelets were subsequently identified by their positivity for CD42b. CD42b-positive events (platelets) were then analyzed for PAC-1 binding by setting a suitable gate (“GPIIb/IIIa act”). Gate position was verified using a control sample of platelets activated with TRAP-6 (final concentration 6 µM, 10 minutes at room temperature). PAC-1 binding was quantified as % of CD42b-positive events that fell into the “GPIIb/IIIa act” gate.

| **Spot Number** | **Protein name** | **Uni-Prot Number** | **Gene Name** | **MW [kDa]** | **pI** | **CV %** |
| --- | --- | --- | --- | --- | --- | --- |
| 2332 | Tropomyosin α-4 chain | P67936 | TPM4 | 28.5 | 4.67 | 8.9 |
| 2385 | 14-3-3 epsilon | P62258 | YWHAE | 29.2 | 4.63 | 9.2 |
| 2533 | 14-3-3 zeta/delta | P63104 | YWHAZ | 27.7 | 4.73 | 7.5 |
| 2518 | 14-3-3 eta | Q04917 | YWHAH | 28.2 | 4.76 | 12.4 |
| 1560 | ATP synthase subunit β, mitochondrial | P06576 | ATP5F1B | 56.6 | 5.26 | 8.6 |
| 2169 | Guanine nucleotide-binding protein G(I)/G(S)/G(T) subunit β-1 | P62873 | GNB1 | 37.4 | 5.60 | 6.8 |
| 2728 | Growth factor receptor-bound protein 2 | P62993 | GRB2 | 25.2 | 5.89 | 6.0 |
| 2663 | Peroxiredoxin-6 | P30041 | PRDX6 | 25.0 | 6.00 | 9.1 |

*MW: molecular weight; pI: isoelectric point; FC: fold change; CV: coefficient of variation*

**Supplementary Table 2. Characterization of low biological variation proteins in platelets to eliminate any disturbing quantity effects of plasma contamination in the COVID-19 platelet proteome data set.** The biological variation of these proteins was calculated from the coefficient of variation of these proteins. To characterize these proteins with low biological variation, the platelet proteome database of patients with lung cancer (n = 19), brain tumors (n = 22) and healthy control persons (n = 41) was used (Ercan et al., 2021). The proteins on this list showed the lowest biological variation from the calculated CVs of the 566 included platelet proteins.


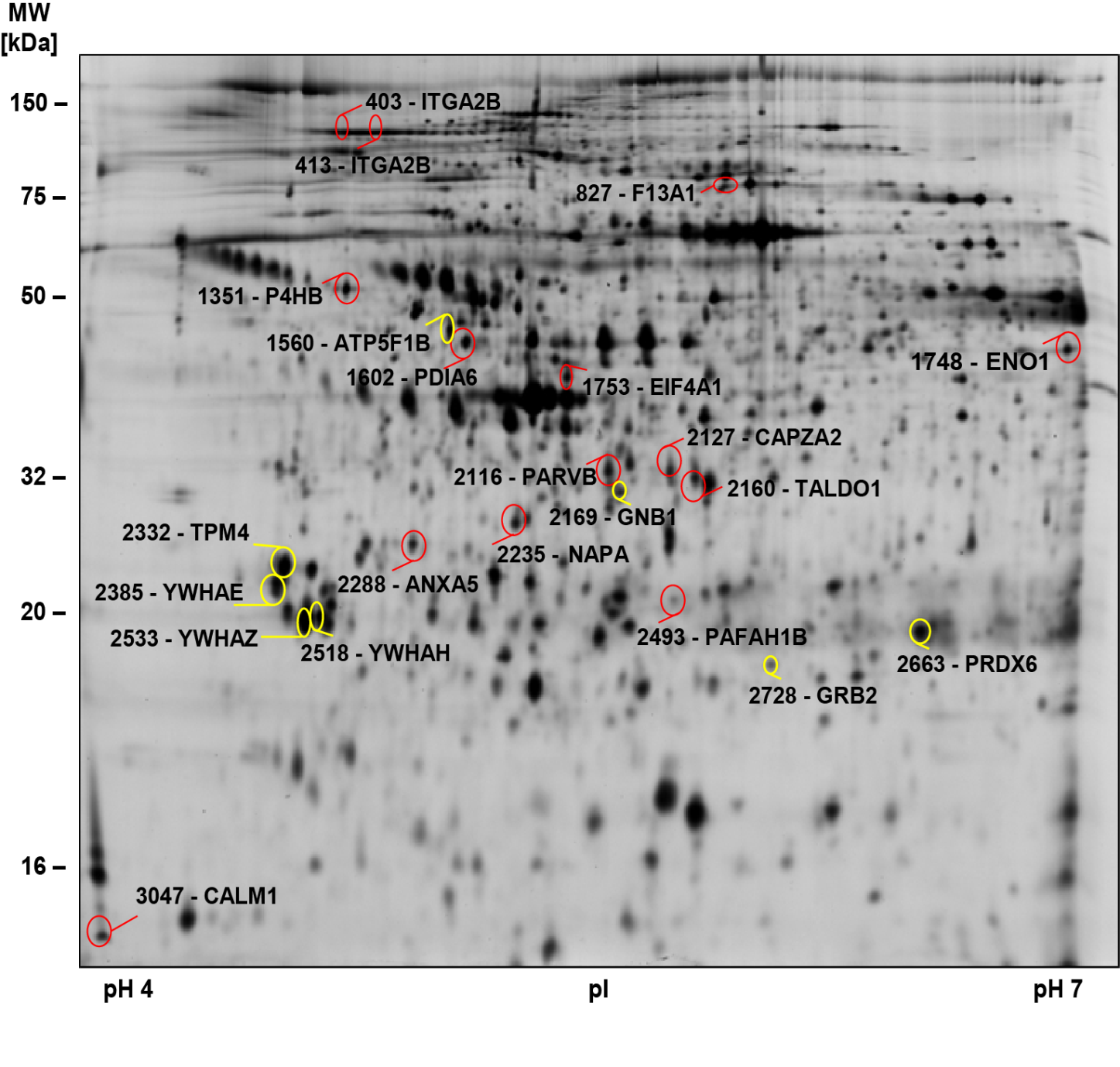


**Supplementary Figure 2. Representative 2D-DIGE gel image with highlighted COVID-19 proteins and low biological variation proteins in the platelet proteome.** Protein spots circled in yellow were used for normalization to eliminate any disturbing quantity effects of plasma contamination. Their corresponding gene names and spot numbers are given in Supplementary Table 2. Significantly altered protein spots of platelets from COVID-19 patients compared to controls identified in the 2D-DIGE-based proteome analysis are circled in red (see also Figure 2, Table 3). Abbreviations: MW – molecular weight; kDa – kilodalton; pI – isoelectric point; 2D-DIGE – two-dimensional differential in-gel electrophoresis.


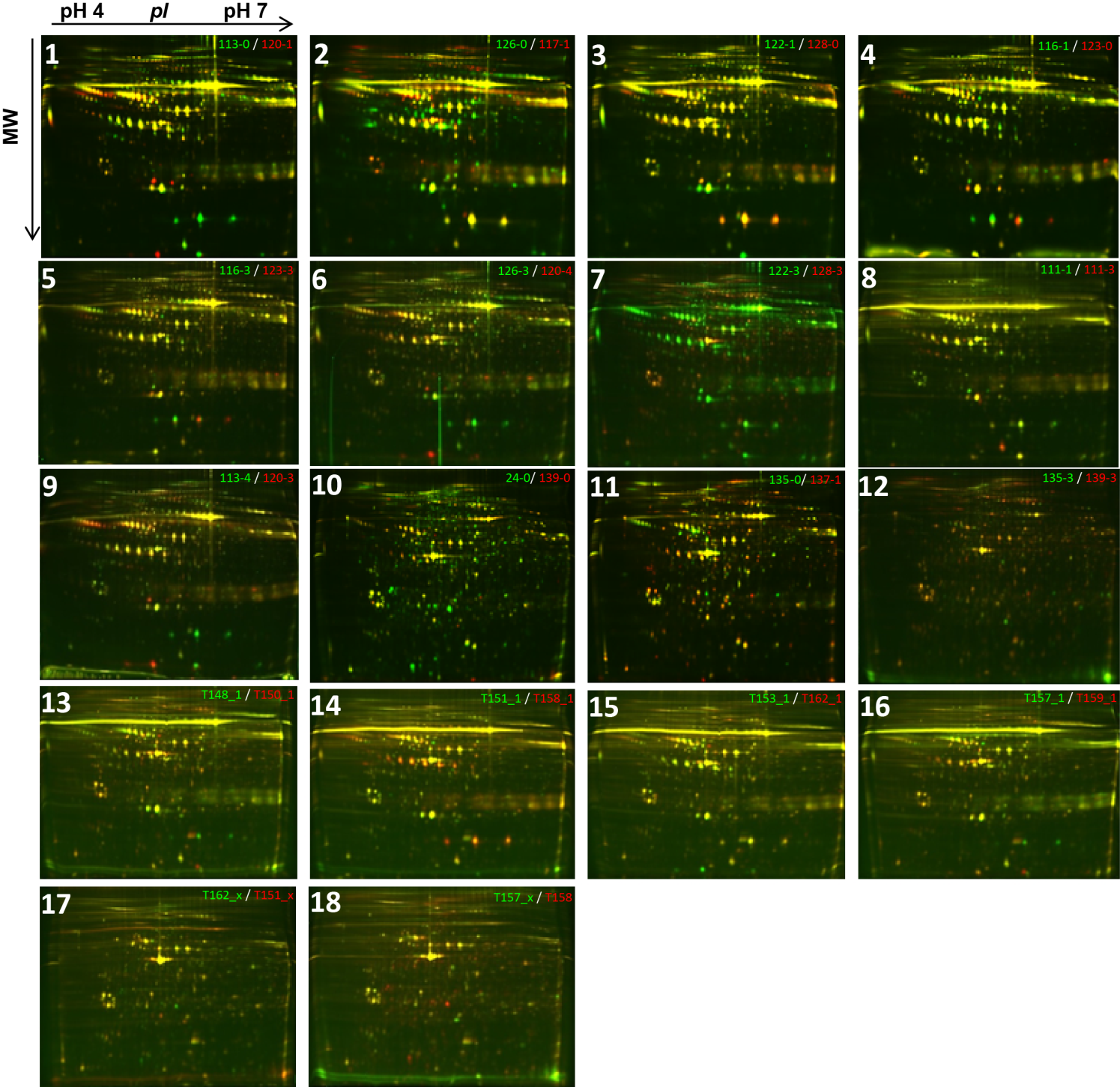


**Supplementary Figure 3. Overview of 2D-DIGE gels from COVID-19 patient platelets vs. healthy controls in the pH range 4-7.** The green spots indicate Cy3-labeled samples, while the red spots indicate Cy5-labeled samples. Yellow spots indicate that the protein spots are equal in both the COVID-19 patients and healthy controls. Abbreviations: 2D-DIGE – two-dimensional differential in-gel electrophoresis; MW – molecular weight; pI – isoelectric point.

**
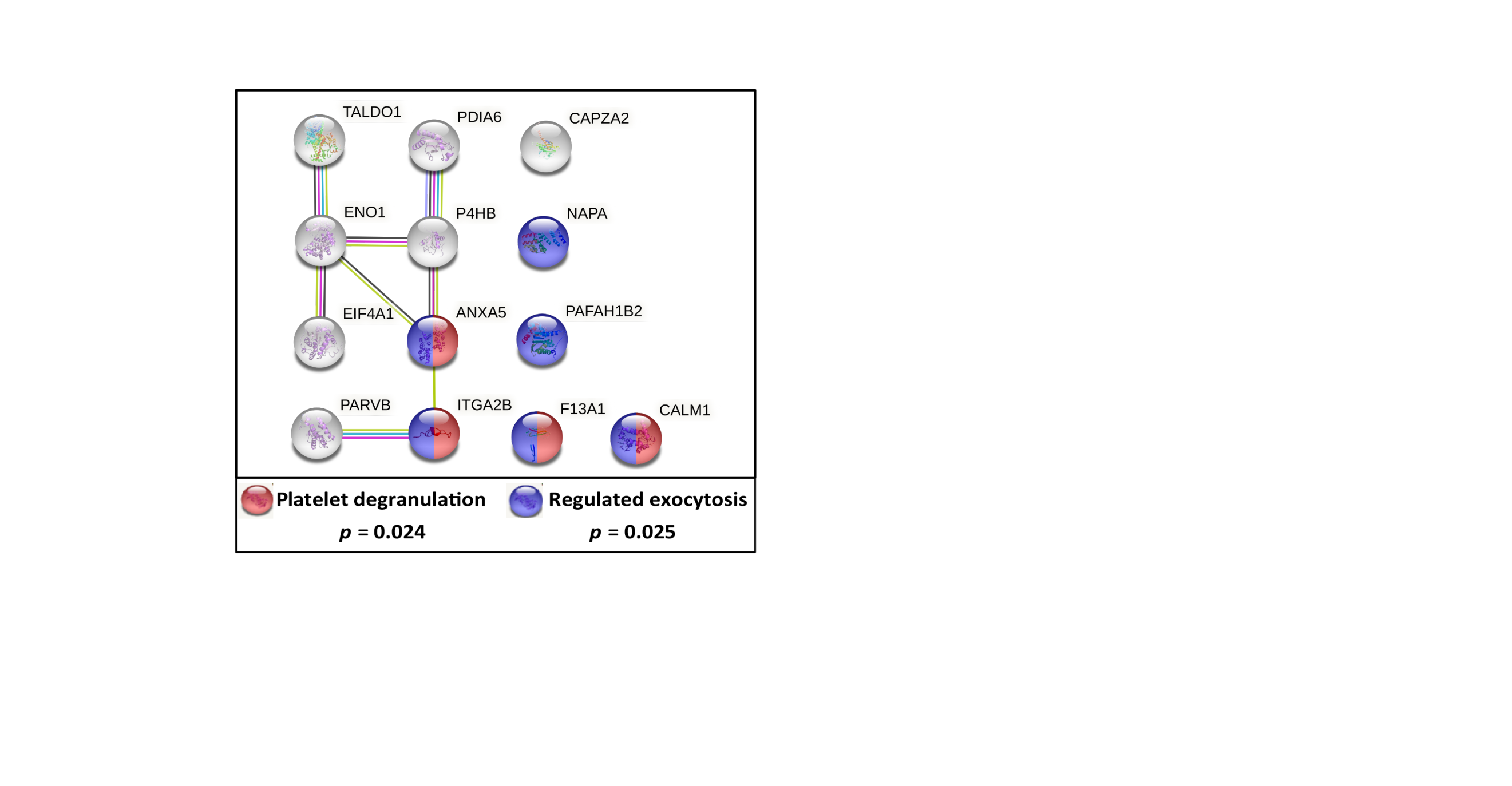
**

**Supplementary Figure 4. Functional association analysis of the COVID-19-related platelet protein.** The network and enrichment analysis from the STRING website shows top pathways obtained upon entering the group of significantly regulated COVID-19-related platelet proteins. The type of interaction is indicated by colored linear slopes; blue: from curated databases, pink: experimentally determined, yellow: text mining, black: co-expression, purple: protein homology. The enrichment graphic depict the most significantly enriched GO Biological Process with platelet degranulation in red and regulated exocytosis in blue. The proteins highlighted in gray were not significantly associated to these quoted functional networks.

**
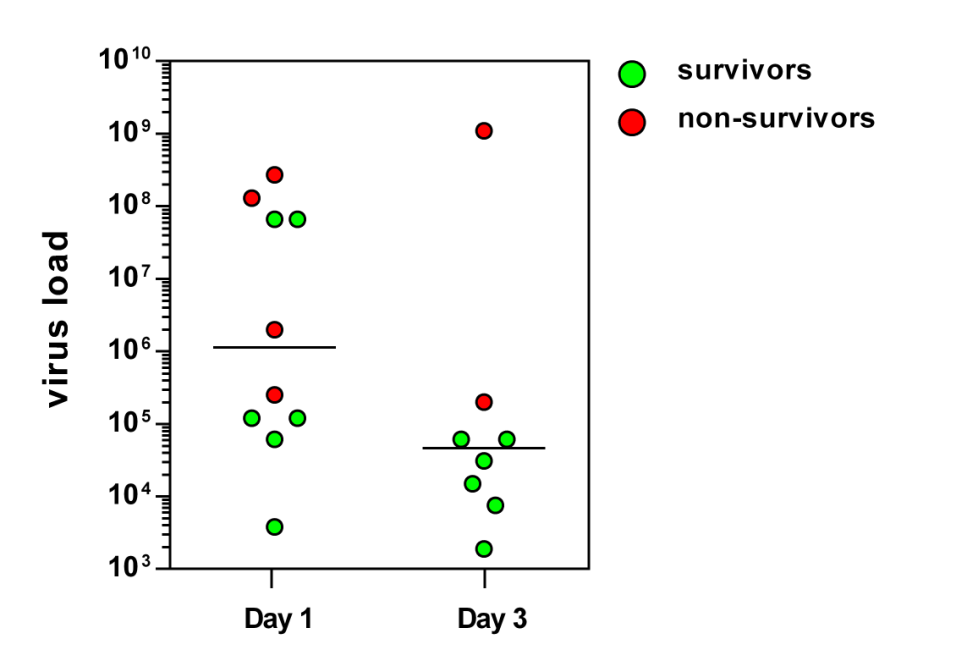
**

**Supplementary Figure 5. Nasopharyngeal virus load in COVID-19 patients.** Scatter dot plot and time course of nasopharyngeal virus load in COVID-19 patients. The nasopharyngeal virus load levels were presented as individual values and means.

**References**

Ercan, H., Mauracher, L.M., Grilz, E., Hell, L., Hellinger, R., Schmid, J.A., Moik, F., Ay, C., Pabinger, I., and Zellner, M. (2021). Alterations of the Platelet Proteome in Lung Cancer: Accelerated F13A1 and ER Processing as New Actors in Hypercoagulability. *Cancers (Basel)* 13.
